# Surgical Approach May Influence Survival of Large-Diameter Head Metal-on-Metal Total Hip Arthroplasty: A 6- to 10-Year Follow-Up Study

**DOI:** 10.1155/2017/4209634

**Published:** 2017-07-24

**Authors:** Chih-Chien Hu, Tsan-Wen Huang, Shih-Jie Lin, Po-Chun Lin, Feng-Chih Kuo, Kuo-Ti Peng, Kuo-Chin Huang, Hsin-Nung Shih, Mel S. Lee

**Affiliations:** ^1^Department of Orthopaedic Surgery, Chang Gung Memorial Hospital, Linkou, Taiwan; ^2^Chang Gung University, Taoyuan, Taiwan; ^3^Department of Orthopaedic Surgery, Chang Gung Memorial Hospital, Chiayi, Taiwan; ^4^Department of Orthopaedic Surgery, Kaohsiung Chang Gung Memorial Hospital, Kaohsiung, Taiwan

## Abstract

Large-diameter head (LDH) metal-on-metal (MoM) total hip arthroplasty (THA) has lost popularity because of metal allergy or ALTRs (adverse local tissue reactions) in the past decade. Whether the surgical approach may influence the survival of LDH-MoM-THA has not been reported. From 2006 to 2009, we performed 96 LDH-MoM-THAs on 80 patients using an in situ head-neck assembly technique through a modified Watson-Jones approach. With a mean follow-up of 8.4 years (range, 6.3–10.1 years), the implant survival rate was 100%. All patients were satisfied with the results and the Harris Hip Score improved from 52 points to 98 points. No ALTRs were found, but 17.7% of the 96 hips (17 adverse events) experienced adverse events related to the cup, including 5 cases of outlier cup malposition, 11 cases of inadequate cup seating, and 1 acetabular fracture. The tissue tension that was improved by a muscle-sparing approach might lessen the chance of microseparation or edge-loading that is taken as the major risk for early implant failure. Further investigation of whether these LDH-MoM-THAs would fail or not would require a longer follow-up or even retrieval analysis in the future.

## 1. Introduction

Large-diameter head (LDH) metal-on-metal (MoM) total hip arthroplasty (THA) has lost popularity recently because the potential advantages of low wear rate, wider range of motion, and lower incidence of dislocation have been overtaken by the ominous complications of metal allergy or ALTRs (adverse local tissue reactions) [1–3]. Higher failure rates of LDH-THA have been reported from the national joint registry database of England and Wales [4] and another multinational study [5]. Although the inferior survivorship is confirmed by systemic review of many studies, the underlying reasons for the higher failure rates are yet to be established [1].

From the technical perspective, more soft tissue dissection is required for LDH-THA because the implantation and reduction of a large femoral head are not easy when using a small wound. In an analysis of 2907 hard-on-hard bearing THAs, Porat et al. reported the incidence of revision due to surgeon-related malposition or subluxation could be as high as 26% [6]. The failure mechanisms are likely related to several factors, including rim contact, impingement, edge-loading, and sliding distance [7, 8]. Edge wear associated with microseparation and malposition of components is recognized as an important risk factor that can lead to metallosis, accelerated wear, ATLRs, and early failure of MoM THAs [9–14].

Severe wear in MoM bearings can be attributed to microseparation and its associated rim contact and edge-loading [15]. The magnitude of microseparation is a critical factor in determining the severity of contact during edge-loading [15]. Inadequate soft tissue tension may contribute to large magnitude of microseparation of hip joint during a gait cycle, for which a translation of the head relative to the cup occurs leading to contact at the superolateral rim of the cup [16, 17]. This contact leads to a narrowed contact area and highly concentrated contact stress resulting in elongated pits and scratches causing substantially rougher wear surfaces and higher wear rate [18]. Less soft tissue dissection through the use of muscle-sparing approaches theoretically could increase soft tissue tension and therefore decrease the chance of edge-loading caused by subluxation during gait cycles. However, the less invasive, muscle-sparing approaches are technically difficult in LDH-THA [19].

The modified Watson-Jones approach is a muscle-sparing technique that uses the tissue interval between the hip abductors and tensor fascia lata for surgery [20, 21]. We hypothesized that the surgical approach may influence the survival of LDH-THA. The purpose of this study was to analyze the clinical and radiological outcomes and survival rate of LDH-THA using the modified Watson-Jones approach. Secondary aims were to review the LDH-THA surgical technique and the potential benefit or difficulties related to the surgical approach

## 2. Materials and Methods

A registry database of LDH-THAs performed using a modified Watson-Jones approach between 2006 and 2009 was reviewed after IRB approval (number 99-3737B). Durom cup, Metasul LDH, and VerSys Fiber Metal Taper stem (Zimmer, Warsaw, IN, USA) were used with the 96 hips (80 patients). A single surgeon (MSL) who was experienced with the approach performed all surgeries. Two independent investigators analyzed the clinical and radiographic outcomes and survival rates of all patients. Hip function and clinical outcomes were assessed on the basis of the Harris Hip Score (HHS) obtained before the operation and at 6 weeks, 3 months, and 6 months and annually thereafter. Hospital course, surgical difficulties, complications, and adverse events were reviewed.

### 2.1. Surgical Technique

**Figure 1:**
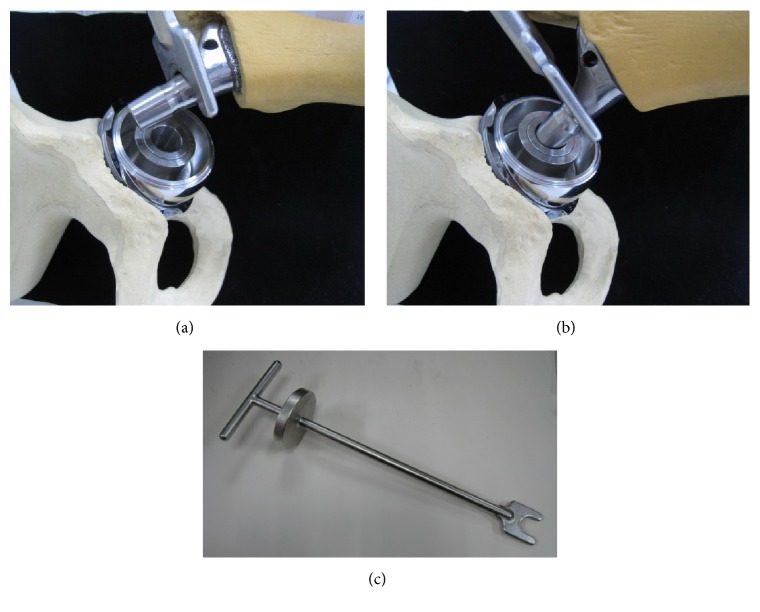
The in situ head-neck assembly technique. (a) A “reduction fork” mounted on the stem neck facilitates the procedure. (b) Engaging the stem neck with the head adaptor. (c) The specially designed “reduction fork.”

The modified Watson-Jones approach described by Bertin and Röttinger was done through the tissue interval between the tensor fascia lata and the gluteus medius, thus avoiding the cutting of any muscles [20]. Special instruments including dog-legged reamer and broach handle were used to accommodate the small incision. After the cup and stem were press-fitted, a trial head with the shortest neck adapter was put into the cup, followed by a technique of in situ head-neck assembly using a hook mounted on the stem neck to control its position, for the engagement of the head and neck (Figure 1). A surgical assistant abducted and pulled the hip while the surgeon maneuvered the head and stem in a coaxial plane. After checking leg length and joint stability, a neck adaptor of ideal length connected to the LDH was put into the cup with its equator facing upward and outward. In situ stem-head assembly was performed and the stability of the taper connection was tested by manually pushing the edge of the LDH under traction.

### 2.2. Radiographic Analysis

Standard pelvis radiographs were obtained for the cup abduction angle, adequate seating of the Durom cup, stem alignment, and canal-filling ratio. In accordance with the literature, outlier malpositioning of the cup was defined as an abduction angle of more than 55° or less than 35° [10–12, 22]. Inadequate seating of the Durom cup was defined as a more than 3 mm gap. Stem alignment was measured as the angle between the long axis of the femoral stem and the anatomical axis of the femur on an anteroposterior radiograph. The canal-filling ratio on the anteroposterior radiograph was calculated by dividing the width of the stem by the inner cortical width at 5 cm distal to the lesser trochanter.

### 2.3. For Potential Adverse Tissue Reactions

If there were any doubt for the potential adverse tissue reactions, serum ions levels of cobalt and chromium were checked. In addition, CT scan or MRI study will be arranged. Symptomatic groin pain, thigh pain, asymptomatic swelling or bulging mass over the hip, or any unusual signs such as hearing impairment, tinnitus, blurring vision, skin itching, or squeaking of the joint were specifically assessed.

## 3. Results

All 96 hips were followed up for clinical and radiographic evaluation. The mean duration of follow-up was 8.4 years (range, 6.3 years to 10.1 years). There were 58 men and 22 women, with a mean age of 49.7 years (range, 27 to 82 years) and a mean body mass index of 25.8 (range, 18.8 to 41.8). The primary diagnosis was osteonecrosis of the femoral head in 55 patients, osteoarthritis in 25 patients.

The mean operating time was 143 minutes (range, 80 min to 234 min); mean amount of blood loss was 448 mL (range, 150 mL to 1200 mL); average wound length was 9 cm (range, 5.5 cm to 16 cm); and mean hospital stay was 5 days (range, 3 days to 11 days). Radiographic evaluation showed that the cup abduction angle was 46.3°  ± 5.9° (range, 34° to 68°) at the initial examination and 46.2°  ± 5.9° (range, 33° to 68°) at the latest follow-up. The canal-filling ratio was 97.6%  ± 3.3% (range, 84% to 100%). The stem alignment was valgus 0.5°  ± 1.8° at the initial examination and valgus 0.4°  ± 1.6° at the latest follow-up. There were 5 outlier malpositioned cups (4 cups with a greater than 55° abduction angle and 1 cup with a 34° abduction angle). During serial follow-ups, no migration or loosening of the cup was found, including the 5 malpositioned cups. In 1 case, there was stem subsidence of 0.5 cm, but it was stabilized, and the patient remained asymptomatic during the follow-up period. The other 95 stems were well-fixed with no visible subsidence or change in position. The average amount of increase in leg length was 1.4 ± 3.5 mm on the affected side; however, none of the patients complained of noticeable inequality in leg length.

**Figure 2:**
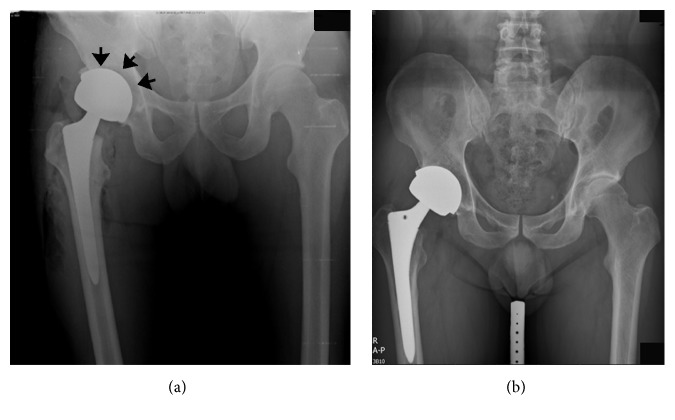
Inadequate seating of the Durom cup in a patient with femoroacetabular impingement. (a) A gap can be seen between the cup and the acetabular floor (arrow). (b) The gap was filled with bone trabeculae at the follow-up examination.

Immediately after the operation, 11 hips (9 patients) were found to have inadequate seating of the Durom cup to the acetabular floor, with a gap of >3 mm behind the shell. However, at the most recent follow-up, all of the 11 inadequately seated cups were still well-fixed without migration or change in position. The gaps between the metal shell and the acetabulum were filled with bone trabeculae (Figure 2). All patients were satisfied with their results. The HHS improved from 52 points (range, 23 points to 78 points) preoperatively to 98 points (range, 88 points to 100 points) at the final follow-up. Six weeks after the operation, 89% of the patients had an HHS >90 points, and this proportion increased to 96% of the patients after 3 months.

### 3.1. Complications

**Figure 3:**
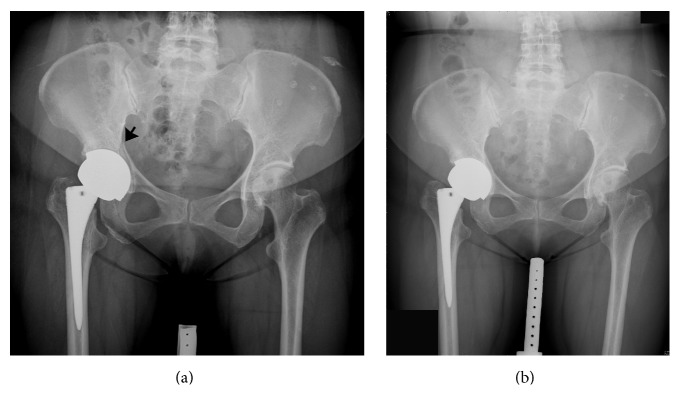
Intraoperative fracture of the acetabulum occurred in 1 hip. (a) The fracture was minimally displaced (arrow). (b) It was united 10 months after the index surgery.

In the study cohort, the Durom cup was associated with 17 adverse events (17.7% of the 96 hips) including 5 malpositioned cups, 11 inadequately seated cups, and 1 intraoperative acetabular fracture (caused by forceful impaction of the Durom cup). The stability of the Durom cup and the fracture were checked. The fracture was managed with protected weight-bearing, and bony union was observed during the follow-up period (Figure 3). The Versys Fiber Metal Taper stem was associated with 3 adverse events (3.1% of the 96 hips). Intraoperative hairline femoral fracture during stem implantation was found in 2 hips that were treated with cerclage wires. Subsidence of femoral stem was found in 1 hip. The femoral stem subsided initially but stabilized at 12 weeks postoperatively. In the final analysis, all femoral stem showed stable osteointegration without evidence of further migration. There were no radiolucent lines at the prosthesis-bone interface and no pedestal formation in any stem.

ALTR was suspected in 1 patient who had intermittent soreness at the hip joint for 1 year postoperatively. A metal suppression CT scan revealed no evidence of local tissue necrosis or fluid accumulation around the hip joint. The serum cobalt level was 0.8 *μ*g/L (normal reference < 1 *μ*g/L) and the serum chromium level was 0.3 *μ*g/L (normal reference < 0.6 *μ*g/L). The patient was informed about the possibility of metal allergy and was followed intensively. Despite these adverse events, all patients were satisfied with their surgical outcomes. No revision or reoperation was performed during the follow-up period.

## 4. Discussion

To our knowledge, this is the first study to report the use of a muscle-sparing approach to perform LDH-THA. We used an in situ head-neck assembly technique to overcome the problem of the small incision and limited tissue dissection hindering the reduction of a LDH. This in situ head-neck assembly technique is easier than the commonly used technique of assembling the head-neck prior to reduction, because the trunnion length of the neck is shorter than the travel distance required for the reduction of a LDH. Reduction after head-neck assembly in a small wound carries the risk of implant fixation failure or periprosthetic fracture if the LDH is jammed into the tissue or onto the edge of the cup. The LDH surface may also be scratched by the sharp edge of the cup during the reduction process. None of the 96 LDH-THAs had fixation failure or periprosthetic fracture related to the technique. Two periprosthetic femoral fractures were encountered during press-fitting of the stems and were fixed stably with cerclage wires before the in situ assembly of the head-neck.

In a study on 180 patients, Long et al. reported that the failure rate of the Durom cup was as high as 23%. Within 2 years after implantation, 29 of the 180 patients (16%), that is, 30 of the 206 hips (15%), required revision surgery for loosening of the Durom cup. Twelve of the remaining 151 patients had a poor or fair clinical grade and/or an HHS of <70 points; these cases were considered to be clinical failures [14]. In a series of 199 THAs (185 patients) performed in the United Kingdom with a mean follow-up of 62 months, the cumulative survival rate was 92.4% at 5 years. After taking into account the patients awaiting surgery, the revision rate would be 15.1%, with a cumulative survival of 89.6% at 5 years [12]. In another study of 100 consecutive THAs performed using the Durom cup, Berton et al. found the survival rate to be 92.4% after 4.8 years of follow-up. When aseptic revision of the component was used as an endpoint, the cumulative survival rate was found to be 94.3% [14]. These authors concluded that the Durom cup should not be used because of its unacceptably high failure rate. The US Food and Drug Administration (FDA) announced a class 2 recall of the Durom cup in September 2008 (recall number Z-2418-2008), which resulted in suspension of the use of this product in the United States. In contrast, with an average follow-up of 8.4 years, we had 100% survival rates of the 96 Durom cups and no ALTRs in all hips. All patients were satisfied and had excellent functional results. We believe that our differing results could not be attributed to constitutive or ethnic differences in our patients, compared to other reports. We thought the difference may be related to the different surgical approach and techniques we used.

Microseparation during a gait cycle leads to contacts between the head and the superolateral rim of the cup and causes severe contact stress and substantially elevated wear [15–18]. The high contact stress critically depends on the magnitude of microseparation. It is suggested that proper soft tissue tension should be considered to avoid microseparation to ensure low wear performance [15]. Since the tissue tension and stability of the LDH-THAs were enhanced by using the muscle-sparing approach and the in situ head-neck assembly technique, the chance of microseparation and edge-loading could be reduced theoretically. In addition, use of the technique obviated scratching on the surface of the LDH and jamming on the edge of the Durom cup during reduction and reduced the abrasive wear on the bearing surface.

In Long et al.'s study, the mechanical characteristic of the specific cup design of the Durom cup may have hindered reliable cup seating [13]. Berton et al. also found an intolerable high incidence (35%) of radiographic visible gap measuring up to 7 mm in depth in their patients [14]. Although we observed gradual bone filling behind the cups in our patients, this technical fault may be inherent and should be a significant concern. The Durom cup is designed as a low profile, truncated hemispheric cup that subtends an angle of 165°. During surgery, the acetabulum is commonly overdeepened through use of the hemispheric reamer. While implanting the cup, the circumferential equatorial fins of the Durom cup may engage with the bone and prevent the cup from seating onto the acetabular floor if the periphery of the acetabulum is sclerotic. Often, surgeons use excessive force to press-fit the Durom cup and ensure adequate seating of the cup on the acetabular floor. Some surgeons have suggested that performing a real-time radiographic examination during surgery may prevent this complication. In our study, 1 acetabulum was fractured during press-fitting of the Durom cup, due to exceeding the amount of force required for implantation. When an inadequately seated Durom cup is observed on fluoroscopy, forceful implantation of the cup without knowledge of the factors hindering correct cup positioning may lead to fracture of the acetabulum. In all, 17.7% of the 96 hips experienced adverse events associated with the Durom cup, which was unacceptably high. These adverse events were all technically related, including inadequate cup seating in 11 hips, malposition in 5 hips, and intraoperative acetabular fracture in 1 hip. We abandoned the use of the Durom cup with our patients in view of these complications.

Elevated serum metal ion levels after LDH-THAs have been reported to be associated with a 32% to 39% incidence of pseudotumor formation [23, 24]. Though not statistically significant, the minimally invasive approach had a 55% incidence of pseudotumor formation while the classical approach had a 45% incidence [23]. Aside from the edge-loading that provokes accelerated wear, mechanical wear or corrosion of the taper has been regarded as a concern for early failure [25–28]. ALTRs elicit local tissue inflammation and soft tissue damage such as bone necrosis or detachment of the muscles from around the femur, which may cause dislocation of the prosthesis [8, 29]. We closely followed our patients and shared information on early failure, elevated serum metal ion levels, and pseudotumor formation with LDH-MoM-THAs that had been reported in the literature. Only 1 of our patients was suspected to have had an adverse reaction to the MoM bearing. Complete radiological study and serum metal ion levels were all proven to be negative for the diagnosis of excessive wear, synovitis, or pseudotumor formation.

There were limitations to this study that should be pointed out. First, we did not perform routine ultrasound, computed tomography, or magnetic resonance imaging for our patients. However, no abnormal signs related to the MoM bearing were detected using a comprehensive physical examination and radiological study. Second, the patient cohort was small and we did not include a comparison group. A comparison group that was operated by using a different surgical approach may clarify the influences of surgical approaches on implant survival. Third, the study was not a prospective randomized-controlled design, so the exact benefits of different surgical approaches could not be concluded. Also, it was not possible to test the hypothesis since the Durom cup had been recalled and such a study would be deemed unethical based on the accumulated evidence of its high failure rates when used with LDH-MoM-THA in the literature and joint registry database. Finally, since we did not routinely measure patients' serum metal ion levels as part of the study protocol, we can not elaborate on the relationship between our technique and serum metal ion levels. It would be of interest to compare the clinical results especially the metal ion levels in our patients with other patients operated by different surgical approach. Such study could provide further evidences to better understand the underlying mechanisms related to the high failure and low survival rates in LDH-THA.

In summary, the 96 LDH-MoM-THAs using a muscle-sparing approach and an in situ head-neck assembly technique were thoroughly followed up. We achieved 100% implant survival with a mean follow-up of 8.4 years. However, we abandoned the use of the Durom cup because of the high incidence of technical difficulty (17.7%) related to it. We had no instances of ALTRs, pseudotumors, or accelerated wear on the MoM bearing. We attributed our exceptionally good results to the enhanced joint stability resulting from use of the muscle-sparing surgical techniques. However, a longer follow-up or even retrieval analysis in the future would be required to further investigate whether these LDH-MoM-THAs would fail or not.
